# Isolated psoas abscess caused by *Mycobacterium tuberculosis*: A rare case report

**DOI:** 10.1002/ccr3.5823

**Published:** 2022-05-27

**Authors:** Mahtab Vasigh, Mohammadreza Karoobi, Mahnaz Montazeri, Golnaz Moradi, Hoda Asefi, Abolfazl Gilani, Seyed mostafa Meshkati yazd

**Affiliations:** ^1^ 48439 Department of Surgery Tehran University of Medical Sciences Tehran Iran; ^2^ 48439 Department of Infectious Diseases Tehran University of Medical Sciences Tehran Iran; ^3^ 48439 Department of Radiology Tehran University of Medical Sciences Tehran Iran

**Keywords:** back pain, diagnosis, *Mycobacterium tuberculosis*, percutaneous drainage, psoas abscess

## Abstract

Psoas tuberculosis abscess is very rarely detected primarily without an adjacent vertebral cold abscess. Early diagnosis prevents unnecessary operations and life‐threatening complications.

## Graphical abstract

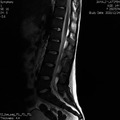

## 1 INTRODUCTION

Psoas abscess is a rare condition that may become a life‐threatening situation. It was described by Mynter in 1881 for the first time.^1^ It can be caused primarily by hematogenous or lymphatic infective spread from a distant lesion or it may be caused as a secondary abscess by direct spread from adjacent anatomical structures.^2^ Primary psoas abscess is a much less common phenomenon.^3^

The most common symptoms include back pain, limp, and fever.^4^ Pain caused by hip extension, the “psoas sign,” may be present in some patients.^5^ The gold standard diagnostic modality is computed tomography (CT). ^6^, ^7^ The mainstay of psoas abscess treatment is antibiotic therapy and CT‐guided percutaneous drainage of the purulent collection^8^; however surgical drainage of the abscess may be inevitable.^9^

*Mycobacterium tuberculosis* is a frequent cause of primary psoas abscess in regions where tuberculosis (TB) is common.^4^ In approximately 20% of all cases of TB in immune‐competent patients (50% in human immunodeficiency virus [HIV]‐positive individuals), extrapulmonary involvement is present, of which the musculoskeletal system is involved in about one tenth of them. It is mostly present as spondylitis, osteomyelitis, or arthritis.^10^, ^11^

However, involvement of skeletal muscle without co‐existing active disease is very rare, with the incidence of primary muscular TB being reported as 0.015% of all cases.^12^, ^13^ Psoas TB usually occurs as an infective spread from adjacent TB focus in the bones or joints, through the rupture and erosion of the walls of an active cold paravertebral abscess.^14^, ^15^

The fact that psoas TB abscess is very rarely detected primarily without an adjacent vertebral cold abscess, and the potential misdiagnosis of a primary TB psoas abscess with soft tissue tumor, hydatid cyst, fungal infection, or hematoma^16^ are the main reasons, we present our experience with such a case. Early diagnosis prevents unnecessary surgical procedures that may lead to further complications.

## 2 CLINICAL CASE

A 14‐year‐old female was admitted to emergency department with severe abdominal pain, nausea, and vomiting that started 2 weeks earlier. She came to Iran from Afghanistan because of her long‐term symptoms and recent exacerbation. She had abdominal pain and night sweats, weakness, and anorexia since last year. Fever and right‐side limp were developed within last 2 months. She was non‐smoker and did not consume any alcohol.

The patient was ill; her temperature was (38.5°C). Blood pressure was 90/60 mmHg, her pulse rate was 90 beats/min, and respiratory rate was 14 breaths/min. The patient's appearance was cachectic. Her chest examinations were clear to auscultation, and her cardiac examination showed regular rhythm without any murmurs, gallops, or rubs. No adenopathy was noted. Abdominal examination revealed normoactive bowel sounds and a right lower quadrant and hypogastric tenderness were present. No mass, distention, or organomegaly was detected. The psoas sign was positive. The hip was fixed in a semi‐flexed position and hip extension was painful. The genital examination was normal.

Laboratory findings revealed a white blood cell count of 18000/μL. The hemoglobin was 8 g/dL, and a low mean corpuscular volume. Electrolytes, blood urea nitrogen, creatinine, and glucose were all within normal limits, as were coagulation studies. Sedimentation rate was 40 mm/h and CRP were 93 mg/L. Blood and urine cultures were negative. Chest x‐ray and ECG study were normal. TSH was within normal limit. Wright and 2 ME, coombs wright, HIV antibody were negative. Echocardiography was normal and ejection fraction was 60%. Tumor markers, including CEA, CA‐125, CA 19–9, were within normal limit.

**FIGURE 1:**
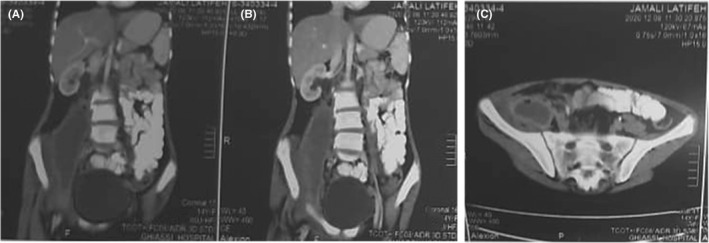
Abdominopelvic CT scan, revealing large collection thorough the entire course of right psoas beginning adjacent to the right kidney up to the right hip. (A), (B) coronal view, (C) axial view

**FIGURE 2:**
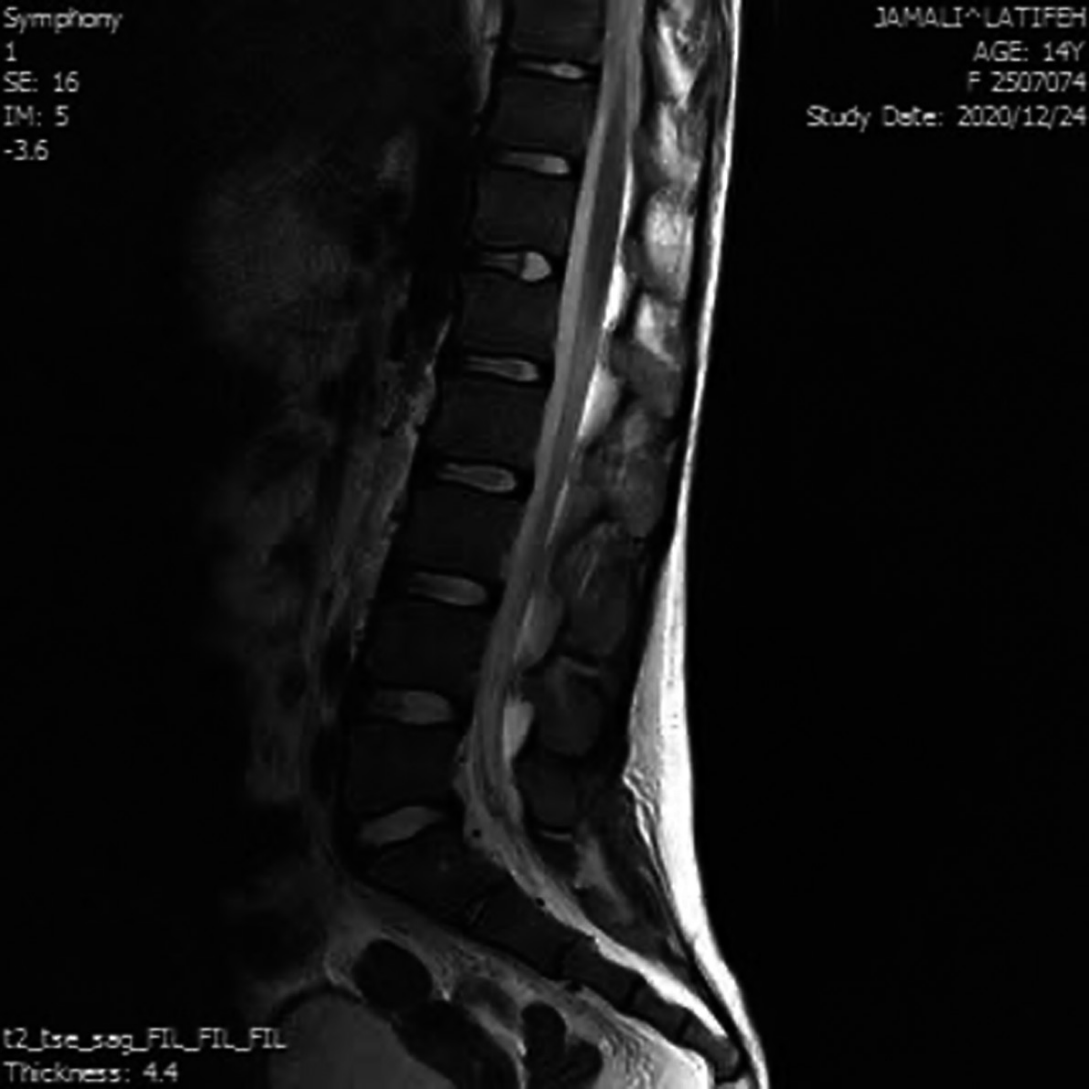
Thoracolumbosacral MRI revealing no evidence of spondylodiscitis adjacent to psoas collection

Abdominopelvic (AP) ultrasound (US) scan showed a collection adjacent to the right kidney, in the area of right psoas muscle, measuring 106X28 mm. AP computed tomography (CT) scan revealed a large collection thorough the entire course of right psoas muscle. Peripheral enhancement was observed after intravenous contrast administration that was suggestive of psoas abscess. Mild right‐sided hydroureteronephrosis was evident (Figure 1). No evidence of spondylodiscitis was revealed on thoracolumbosacral magnetic resonance imaging (MRI) (Figure 2).

US‐guided aspiration of the collection within psoas muscle was performed under aseptic precautions. At once, 300 cc pus was aspirated and 10 0cc/day was drained within the next 5 days thorough the drain which was inserted within the collection. The aspirate was sent to Gram staining, bacterial culture, and PCR analysis of TB. Gram stain revealed numerous white blood cells without bacteria. Aspirate culture revealed no growth of bacteria. TB‐PCR was positive in abscess pus specimen. The patient's treatment was started immediately. Two days after taking anti‐TB drugs, the patient became afebrile. Follow‐up AP CT revealed shrinkage of the collection. All the symptoms subsided. The drain was extracted when the discharge was less than 10 ml/day. Laboratory tests including WBC count and CRP were within normal range at the time of her discharge. Anti‐TB medications continued.

## 3 DISCUSSION AND CONCLUSION

The most common cause of primary psoas abscess is *Staphylococcus aureus* (88% of the cases). Streptococci (5%), *Escherichia coli* (3%), and rarely *Brucella* or pneumococci include other causes of primary psoas abscess. Local extension of infection from an adjacent focus result in a secondary psoas abscess. The most common causes of secondary psoas abscess are peritoneal pathologies and spinal infections.^13^ Aside from all these facts, primary psoas abscess without any presence of an infective focus, as seen in our case, is very rare in the literature.^13^

*M*.* tuberculosis* has been the predominant pathogen causing psoas abscess before advancement in anti‐TB treatment regimens, but today, it rarely occurs. TB of the vertebra (Pott's disease) is the most common cause of secondary psoas abscess, in developing countries. Pott's disease can cause a secondary TB psoas abscess in 5% of the cases.^11^

TB psoas abscess as the only manifestation of TB in a patient without any previous predisposing pathology is exceptional. To our knowledge, (Pubmed up to 2021 Abscess, Psoas, Tuberculosis), 9 cases have been reported previously.^11^, ^17^, ^18^, ^19^, ^20^, ^21^, ^22^, ^23^, ^24^

One of which was reported as a postpartum complication in which patient was an afebrile young woman with low back pain which was drained and four weeks after the cavity was reaccumulated and on the second drainage, TB was diagnosed and appropriate antibiotic therapy was commenced.^17^ Another case was reported with non‐specific presentation; however, patient's husband was diagnosed with pulmonary TB 3 years prior. The diagnosis was established after many tests with a 4 weeks delay (due to the rarity of this condition) through a positive Lõwenstein culture. Patient was then treated with drainage and antibiotics.^21^

As in our case, those other reports presented young patients, without any predisposing illness. However, they generally had a good general condition with subacute or chronic symptoms. Our case was admitted with an ill general condition. It was due to her long‐term misdiagnosis. Chest and spine evaluation were normal and the psoas abscess aspirate was the only specimen that yielded positive result on TB ‐PCR. Conclusively, it was considered as primary psoas abscess.

Psoas abscess causes nonspecific symptoms and subtle physical findings; therefore, it is a diagnostic challenge. Plain chest and abdominal radiographs may remain inadequate and AP US scan and cross‐sectional imaging are usually needed.

TB psoas abscess is usually treated with percutaneous drainage and appropriate anti‐TB therapy; however, some cases may require surgical interventions.^12^, ^25^ Our patient responded well to percutaneous drainage and anti‐TB therapy. Although we were going to drain the abscess surgically, diagnosis of TB led to anti‐TB medication. After 48 h, the symptoms resolved and surgery was no longer necessary. If we were not aware of TB, an open drainage could result in the spread of infection into abdominal cavity, increasing the risk of future tubal adhesions and infertility.

In summary, we suggest that when the diagnosis of psoas abscess is established, TB should be considered as a causative agent, even in the absence of a vertebral cold abscess. If TB psoas abscess is confirmed, medical therapy and closed abscess drainage may prevent surgical interventions and late complications.

## AUTHOR CONTRIBUTIONS

MV: Conceptualization and investigation and writing the manuscript. MK: Case presentation section and interpreted the patient data. MM: Evaluation of case reports and writing the manuscript. HA: Obtained consent for participation and publication from patient's parents and writing the manuscript. AG: Helped in drafting the original manuscript. SMMY: reviews/edits of case report write‐up, appropriate source citation. All authors read and approved the final manuscript.

## CONFLICT OF INTERESTS

The authors declare no competing interests.

### ETHICAL APPROVAL

The study was approved by the Ethics Committee of the Tehran University of Medical Sciences. Permission to carry out the study and access patient records was sought from the respective university administrators.

### CONSENT

Written informed consent for publication of her clinical details was obtained from the parents of the patient.

## Data Availability

The datasets used and/or analyzed during the current study are available from the corresponding author on reasonable request and with permission of Research Ethics Committee of School of Medicine‐Tehran University of Medical Sciences.
